# μFlow: A Computational Platform for Microfluidic Hall-Effect Magnetic Bead Detection with Parametric Design Optimization

**DOI:** 10.3390/mi17091042

**Published:** 2026-08-31

**Authors:** Harshitha Govindaraju, Umer Hassan

**Affiliations:** Department of Electrical and Computer Engineering, Rutgers, The State University of New Jersey, 94 Brett Road, Piscataway, NJ 08854, USA; harshitha.govindaraju@rutgers.edu

**Keywords:** microfluidics, Hall effect, magnetic bead detection, biosensor, analytical–numerical framework, design optimization

## Abstract

Microfluidic Hall-effect biosensors detect superparamagnetic bead labels as they flow past a thin-film Hall element in a microchannel. Designing one couples bead magnetization, stray-field distribution, Hall transport, and channel flow across 14 parameters that finite-element solvers explore only at minutes to hours per configuration. We present a coupled analytical–numerical framework for this signal chain: Clausius–Mossotti bead magnetization with a volume fraction correction, a point-dipole stray field, a volume-averaged Hall voltage, Poiseuille transport, and a Johnson–Nyquist and Hooge 1/f noise model, evaluated across 12 sensor presets compiled from the literature, spanning graphene, III–V semiconductors, Si CMOS, bismuth, and topological insulators; any other platform can be defined from user-supplied transport parameters. Benchmarked against a companion COMSOL Multiphysics 6.0 study, the framework reproduces the Hall voltage to within 4.8% at a favorable bead-to-sensor area ratio and deviates by 22% and 15% at off-optimum geometries, consistent with the point-dipole near-field limit at $h/r_{b}=1$. Three design rules follow: a signal-to-noise ridge at sensor widths comparable to the bead diameter ($w^{*}\approx d_{b}$; area ratios 0.4–1.0 at constant voltage, 0.5–2.6 at constant current), matching reported single-bead geometries; a material choice that must be made under an explicit electrical drive constraint; and a sampling-limited flow-velocity window. Predicted signals agree at the order-of-magnitude level with published InAs and Si CMOS experiments. We release the model as a freely accessible, no-install browser implementation with a built-in 2D axisymmetric magnetostatic finite-element (FEM) solver that maps where the dipole approximation degrades.

## 1. Introduction

Microfluidic Hall-effect sensors detect individual superparamagnetic beads by measuring the transient stray magnetic field as labeled analytes flow past a thin-film Hall element embedded in a microchannel floor. The approach has been demonstrated across multiple sensor platforms. Ejsing et al. measured 0.3 μV signals from 2 μm beads with a planar Hall sensor [1]. Mihajlović et al. detected a single commercial 1.2 μm superparamagnetic bead at room temperature with an InAs quantum-well micro-Hall sensor [2] (the measured signal is analyzed in Section 3.2). Shah et al. integrated chemical-vapor-deposition (CVD) graphene Hall sensors ($S_{I}=175\;V\;A^{-1}\;T^{-1}$) into a polydimethylsiloxane (PDMS) microchannel and detected ferrofluid-loaded model beads flowing in protein buffer and diluted blood, with long-term sensor stability characterized in blood plasma [3]. Gabureac et al. detected single superparamagnetic beads with sub-200 nm nanocomposite nano-Hall sensors [4]. The diversity of viable platforms, which now spans graphene [5,6], III–V semiconductors [2,7,8,9], silicon complementary metal–oxide–semiconductor (CMOS) [10], and topological insulators [11], makes material selection nontrivial because the detected signal depends on the coupled interaction of several physics domains.

The design challenge arises from several interacting effects. The magnetization of the bead in the applied field depends on its ferrite content and effective susceptibility through the Clausius–Mossotti relation. The spatial distribution of the bead’s dipolar stray field decays as $1/r^{3}$ and changes sign at lateral offsets. The Hall voltage is produced by averaging this inhomogeneous field over the sensor active volume, so the signal depends jointly on bead size and sensor footprint. The hydrodynamic transport determines the bead trajectory and transit time through the sensing zone via the Poiseuille velocity profile. Commercial finite-element tools such as COMSOL Multiphysics (v6.0; COMSOL AB, Stockholm, Sweden) can model each of these domains, but setting up a coupled magnetostatic–hydrodynamic simulation requires a software license and minutes to hours of computation per configuration. Our companion study used a COMSOL finite-element (FEM) model to characterize three sensor geometries across bead sizes, magnetization levels, and bias conditions [12]. Jebari et al. reported a 3D multiphysics simulation of a microfluidic immunosensor coupling magnetostatic, fluid-dynamic (Navier–Stokes), and electrostatic solvers across multiple geometry variants [13]. Closed-form analytical work covers the individual modules in isolation: point-dipole stray fields [14], planar Hall voltage [15], and Poiseuille flow [16]. The bead-comparable Hall-cross geometry used for single-bead detection by Mihajlović et al. with InAs micro-Hall sensors [17] addresses geometry but not the full signal chain. No freely available model couples these modules into a single, parameterized signal chain that a non-specialist can evaluate without a commercial solver.

This paper presents such a model and the design rules that follow from it. The contributions, in order of priority, are: (i) a coupled analytical–numerical framework for the complete microfluidic Hall-effect bead-detection signal chain, benchmarked against a 3D FEM study: the model reproduces the benchmark Hall voltage to 4.8% at the favorable bead-to-sensor area ratio and to 15–22% at off-optimum geometries, all evaluated at the dipole near-field limit $h/r_{b}=1$; (ii) design rules extracted from the model, including a signal-to-noise-optimal sensor width comparable to the bead diameter ($w^{*}\approx d_{b}$) that is consistent with the bead-comparable sensor geometries used in reported single-bead detections [17], a cross-material comparison resolved by an explicit electrical drive constraint, and a sampling-limited flow-velocity window; and (iii) a freely accessible, no-install browser implementation, with an in-app 2D axisymmetric magnetostatic FEM solver, that quantifies where the point-dipole approximation degrades. The implementation is deployed at https://uflow.studio (accessed on 21 August 2026) and runs in any modern browser without installation, registration, or a license.

## 2. Model and Methods

The model implements the analytical–numerical signal chain benchmarked against our companion COMSOL FEM study [12]; the individual modules are closed-form expressions, coupled through a numerical volume-averaging quadrature, which is why we describe the framework as analytical–numerical. We describe each component, the noise framework, the microfluidic transport, the material database, and the browser implementation that evaluates the model and provides an independent FEM check.

### 2.1. Bead Magnetization

Each superparamagnetic bead of radius $r_{b}$ contains magnetic nanoparticle (NP) cores with core relative permeability $\mu_{r}$ dispersed in a polymer matrix at magnetic volume fraction ϕ. The induced magnetic dipole moment is

(1) $$m=\frac{4}{3}\pi r_{b}^{3}\;\phi \;\chi_{\mathrm{eff}}\;\frac{B_{0}}{\mu_{0}},$$

where $\mu_{0}=4\pi \times 10^{-7}$ T mA^−1^ is the vacuum permeability, ϕ is the volume fraction of the magnetic phase (Section 2.6), and $\chi_{\mathrm{eff}}$ is the Clausius–Mossotti effective susceptibility of the core ferrite material:

(2) $$\chi_{\mathrm{eff}}=\frac{3(\mu_{r}-1)}{\mu_{r}+2}.$$

For magnetite ($\mu_{r}=10$), Equation (2) gives $\chi_{\mathrm{eff}}=2.25$. The factor ϕ in Equation (1) is needed for composite beads: Dynabeads M-280 (Thermo Fisher Scientific, Waltham, MA, USA) contain ∼5% iron oxide by volume (computed from the 118 mg/g iron content reported by Fonnum et al. [18]), so their effective moment is ∼20× smaller than a solid magnetite sphere of the same diameter. For the COMSOL benchmark beads, ϕ=1 (pure magnetite).

This linear magnetization model is valid in the sub-saturation regime ($B_{0}≲200\;mT$ for magnetite NPs with $d_{\mathrm{NP}}\approx 16\;nm$). At higher fields, the NP moments approach saturation and a Langevin ensemble model is required [19,20], with the dynamics of the individual nanoparticle moments under anisotropy and thermal fluctuations treated in Reference [21]; the implementation displays a warning when $B_{0}$ exceeds 200 mT with commercial bead presets. For practical microfluidic assays operating at $B_{0}=$ 20–100 mT, the linear Clausius–Mossotti model is appropriate.

### 2.2. Point-Dipole Stray Field

The induced moment lies along *z*, parallel to the applied field. The axial component of its field at a point displaced from the dipole by (Δx,Δy,Δz) is [14]

(3) $$B_{z}\left(\Delta x,\Delta y,\Delta z\right)=\frac{\mu_{0}}{4\pi }\;m\;\frac{2\Delta z^{2}-\Delta x^{2}-\Delta y^{2}}{{\left(\Delta x^{2}+\Delta y^{2}+\Delta z^{2}\right)}^{5/2}}.$$

The point-dipole form is the leading term of a multipole expansion and loses accuracy when the bead–sensor separation approaches the bead radius ($h/r_{b}∼1$) [14]; the built-in FEM solver (Section In-App 2D Axisymmetric FEM Solver) quantifies this deviation for each geometry. The FEM solver provides a direct comparison between this approximation and a full volumetric magnetostatic solution, so the dipole-model validity can be checked for any on-axis combination of bead size, height, and sensor size.

Equation (3) produces a characteristic spatial signature: $B_{z}$ is positive directly above and below the dipole, crosses zero at $\Delta z^{2}=\left(\Delta x^{2}+\Delta y^{2}\right)/2$, and becomes negative at large lateral offsets. As a bead traverses the sensor along the channel axis, the volume-averaged field traces a bipolar pulse whose amplitude and width depend on the bead–sensor distance and the sensor footprint.

### 2.3. Volume-Averaged Hall Voltage

The Hall voltage depends on the average $B_{z}$ over the sensor volume, not on the surface field at a single point. The model computes this by midpoint quadrature over an $n_{xy}\times n_{xy}\times n_{z}$ grid spanning the sensor volume (width *w*, length *l*, thickness *t*):

(4) $$\langle B_{z}\rangle =\frac{1}{n_{xy}^{2}\;n_{z}}\sum_{i=1}^{n_{xy}}\sum_{j=1}^{n_{xy}}\sum_{k=1}^{n_{z}}B_{z}\left(\Delta x_{i},\Delta y_{j},\Delta z_{k}\right),$$

where each grid point samples Equation (3). The resulting Hall voltage change is

(5) $$\Delta V_{H}=G\;R_{H}\;\sigma \;\langle B_{z}\rangle \;V_{\mathrm{bias}},$$

where $R_{H}$ is the Hall coefficient ($m^{3}\;C^{-1}$), σ is the sensor conductivity ($S\;m^{-1}$), $V_{\mathrm{bias}}$ is the applied bias voltage, and G∈[0.3,1.0] is a geometric correction factor that accounts for current spreading in non-square Hall crosses [15]. For a symmetric Greek-cross geometry, G≈1; for long, narrow Hall bars (l/w>3), *G* approaches the ideal value of 1; for short, wide geometries (l/w<1), *G* can drop below 0.7 [15]. The implementation fixes G=1, since all built-in and user-defined sensor elements are square; for non-square elements, the reported $\Delta V_{H}$ can be scaled by the appropriate *G* from Reference [15]. For the three benchmark designs in Reference [12], $R_{H}=1.25\times 10^{-3}\;m^{3}\;C^{-1}$, $\sigma =1040\;S\;m^{-1}$, and G=1.

For the 12 material presets (Section 2.6), the Hall coefficient is computed from the carrier density. For two-dimensional materials with sheet carrier density $n_{s}$ ($m^{-2}$),

(6) $$R_{H}^{2D}=\frac{1}{n_{s}\;e},\;S_{I}=\frac{1}{n_{s}\;e},$$

where $e=1.6\times 10^{-19}\;C$ and $S_{I}$ is the current-related sensitivity ($V\;A^{-1}\;T^{-1}$). For three-dimensional materials with bulk carrier density *n* ($m^{-3}$) and film thickness *t*,

(7) $$R_{H}^{3D}=\frac{1}{n\;e},\;S_{I}=\frac{R_{H}^{3D}}{t}=\frac{1}{n\;e\;t}.$$

For gate-tunable 2D materials, namely the six gate-parameterized presets of Table 1 (CVD graphene, graphene/hBN, GaAs/AlGaAs, AlGaN/GaN, InSb quantum well (QW), and Bi_2_Se_3_), the carrier density varies with the applied gate voltage $V_{g}$ relative to the charge neutrality point $V_{\mathrm{CNP}}$:

(8) $$n_{s}\left(V_{g}\right)=\sqrt{n_{s,0}^{2}+{\left(\frac{C_{\mathrm{ox}}}{e}\;\left|V_{g}-V_{\mathrm{CNP}}\right|\right)}^{2}},$$

where $n_{s,0}$ is the residual (charge-puddle) carrier density at neutrality and $C_{\mathrm{ox}}$ is the gate oxide capacitance per unit area; the quadrature form gives a smooth crossover from the residual density near the neutrality point to the gate-induced density at large $|V_{g}-V_{\mathrm{CNP}}|$. Reducing $n_{s}$ increases the Hall coefficient but also increases the device resistance and associated Johnson noise, so the gate slider exposes the sensitivity–noise trade-off directly.

The volume averaging in Equation (4) captures the joint dependence on bead size and sensor footprint: the signal falls as the bead is displaced laterally out of the sensor active area. Uddin et al. reported for elliptical planar Hall sensors that the sensitivity increases with sensing area and decreases with film thickness [22].

### 2.4. Noise and Detection Limits

Whether a bead is detectable is set by the signal-to-noise ratio (SNR), not by the Hall voltage magnitude alone. The model implements a noise framework covering the dominant noise sources in Hall sensors: Johnson–Nyquist (thermal) noise and Hooge 1/f (flicker) noise. The sheet resistance of the sensor is

(9) $$R_{□}=\frac{1}{n_{\mathrm{tot}}\;e\;\mu },$$

where $n_{\mathrm{tot}}$ is the total carrier density (written $n_{s,\mathrm{tot}}$ when expressed as a sheet density: $n_{s}$ for 2D presets and nt for 3D presets of film thickness *t*) and μ is the carrier mobility. For a square sensor biased at $V_{\mathrm{bias}}=I_{\mathrm{bias}}R_{□}$, the thermal noise voltage in a measurement bandwidth Δf is

(10) $$V_{\mathrm{th}}=\sqrt{4k_{B}TR_{□}\Delta f},$$

where $k_{B}$ is the Boltzmann constant and *T* is the temperature. The 1/f noise voltage, following the Hooge model [23,24], is

(11) $$V_{1/f}=V_{\mathrm{bias}}\sqrt{\frac{\alpha_{H}}{N}\;\ln\;\left(1+\frac{\Delta f}{f_{\mathrm{op}}}\right)},$$

where $\alpha_{H}$ is the dimensionless Hooge parameter, $N=n_{\mathrm{tot}}A$ is the total carrier count in the sensor active area *A*, and $f_{\mathrm{op}}$ is the operating frequency. The total noise voltage and detection metrics are

(12) $$V_{n}=\sqrt{V_{\mathrm{th}}^{2}+V_{1/f}^{2}},\;\mathrm{SNR}=\frac{|\Delta V_{H}|}{V_{n}},\;B_{\min}=\frac{V_{n}}{\sqrt{\Delta f}\;\left|S_{I}\right|\;I_{\mathrm{bias}}},$$

where $B_{\min}$ is the noise-equivalent magnetic field spectral density (per $\sqrt{\mathrm{Hz}}$): the band-averaged noise voltage density $V_{n}/\sqrt{\Delta f}$ referred to the field through the current-related sensitivity. Because $V_{n}$ integrates the 1/f density over $\left[f_{\mathrm{op}},f_{\mathrm{op}}+\Delta f\right]$, $B_{\min}$ is a band average; for 1/f-dominated configurations, the spot density at $f_{\mathrm{op}}$ is up to ∼20% higher at the conditions of Section 3.3. All noise metrics depend on the electrical drive constraint (constant voltage, constant current, or constant power), which is stated explicitly for every comparison in Section 3.3. A bead is considered detectable when SNR>3. The time-domain analyses of Section 3.5 and Section 3.6 instead count a bead as detected when its peak $|\Delta V_{H}|$ exceeds a stated voltage threshold, the criterion implemented in the simulator.

The Hooge parameter $\alpha_{H}$ varies by several orders of magnitude across sensor platforms, from $∼10^{-5}$ for high-quality encapsulated graphene to $∼10^{-2}$ for disordered thin films (Section 3.3); for CVD graphene Hall elements, $\alpha_{H}$ is of order $10^{-4}$ [24]. This material-dependent noise floor shapes the cross-material comparison: a platform with high mobility and high $\alpha_{H}$ may produce a weaker SNR than a lower-mobility platform with superior noise performance, a trade-off that $\Delta V_{H}$ alone cannot capture. The model accepts a user-specified $\alpha_{H}$ and computes the noise floor, SNR, and $B_{\min}$ alongside $\Delta V_{H}$ for every configuration, enabling noise-aware design optimization when published noise characterization data are available for the platform of interest.

### 2.5. Microfluidic Transport

Bead trajectories follow Poiseuille flow in a rectangular microchannel. For a channel with height *H* and width W≫H (aspect ratio >10:1, so that sidewall drag is negligible), the streamwise velocity at vertical position *y* is

(13) $$v\left(y\right)=\frac{3}{2}\;\bar{v}\left[1-{\left(\frac{2y}{H}-1\right)}^{2}\right],$$

where $\bar{v}$ is the mean flow velocity. Beads at the channel center (y=H/2) travel at $1.5\bar{v}$, while those near the walls approach zero velocity. Hewlin and Edwards used this parabolic profile to model particle trajectories in a Y-shaped microfluidic channel, confirming that laminar flow assumptions hold for Reynolds numbers below ∼1 in channels of comparable dimensions [16]. Khashan et al. showed that coupling Poiseuille flow with magnetophoretic body forces enables selective sorting of labeled versus unlabeled particles in similar geometries [25].

The model distinguishes two height parameters: *h*, the bead-center distance above the sensor surface, and *H*, the channel height. Only *h* enters the dipole stray-field formula and therefore directly drives $\Delta V_{H}\propto 1/h^{3}$; *H* does not appear in the signal formula but bounds where *h* can sit (geometric constraint $r_{b}\leq h\leq H-r_{b}$) and shapes the velocity profile via the y/H dependence. In fixed-height mode, all beads share the same *h*, which isolates the influence of sensor, bead, or material parameters. In range mode, each bead’s *h* is drawn from a uniform distribution over a user-set interval within the allowed range, so the per-bead signals span the $1/h^{3}$ decay, and a constant downward drift ($∼0.3\;\mu m\;s^{-1}$) pulls beads toward the sensor surface, approximating magnetic capture. The uniform sampling is a deliberately conservative baseline: in a flowing channel, the equilibrium height distribution is biased toward the floor by sedimentation (Stokes velocity $v_{s}=\frac{2}{9}\Delta \rho gr_{b}^{2}/\eta \approx 2\;\mu m\;s^{-1}$ for a 2.8 μm M-280 Dynabead in water) over channel residence times, which concentrates beads where $1/h^{3}$ signals are strongest, so detected SNR in practice tends to exceed the uniform-range prediction. The bead position is updated each frame as x(t+Δt)=x(t)+vΔt, where v=v(y) from Equation (13) in range mode and $v=\bar{v}$ in fixed-height mode; adaptive sub-stepping caps the displacement per sub-step at $0.4\;w_{\mathrm{sensor}}$ so that the narrow signal peak is not stepped over between frames. This rendering-loop rule governs only the interactive animation; for the flow-window analysis (Section 3.5), the readout is modeled explicitly as a sampled acquisition at a stated rate $f_{s}$, decoupled from the animation loop.

### 2.6. Material Database

The model carries 12 Hall sensor material presets spanning graphene, III–V semiconductors, Si CMOS, bismuth, and topological-insulator families (Table 1). Each preset specifies the carrier mobility μ, the carrier density ($n_{s}$ for 2D or *n* for 3D), and the dimensionality. All parameter values were extracted from peer-reviewed publications as indicated. For the six gate-parameterized presets (CVD graphene, graphene/hBN, GaAs/AlGaAs, AlGaN/GaN, InSb QW, and Bi_2_Se_3_), a gate voltage can be applied via Equation (8) to shift the operating point along the sensitivity–noise trade-off curve. The 12 presets are a literature-derived starting set rather than a limit of the model: a platform not listed can be entered directly by supplying its mobility, carrier density, dimensionality, and Hooge parameter, and is then treated identically to a preset throughout. Since $R_{H}\sigma =\mu$ (Equations (5)–(7)), the Hall voltage at fixed bias voltage is proportional to the mobility, $\Delta V_{H}=G\;\mu \;\langle B_{z}\rangle V_{\mathrm{bias}}$, while at fixed bias current, it is set by the carrier density, $\Delta V_{H}=G\;S_{I}I_{\mathrm{bias}}\langle B_{z}\rangle$ with $S_{I}\propto 1/n_{s,\mathrm{tot}}$; the database therefore supports cross-platform comparison under an explicitly stated drive constraint.

Table 2 lists six representative presets from the software’s bead library, which additionally includes two sub-micron presets (nanomag-D 250 and SpeedBeads). The first three are commercially available superparamagnetic beads widely used in immunoassay and cell sorting; the last three match the magnetite beads used in the COMSOL benchmark study [12].

**Table 1:** Material property database. μ: carrier mobility; $S_{I}=1/\left(n_{s}e\right)$ for 2D materials; $R_{H}=1/\left(ne\right)$ for 3D. All values at room temperature. Abbreviations: QW, quantum well; MBE, molecular-beam epitaxy; TI, topological insulator.

Material	μ (cm^2^/Vs)	$S_{I}$ or $R_{H}$	Refs.
*Graphene (2D)*			
CVD graphene	5000	1250 V/AT	[23,24]
Graphene/hBN	40,000	5700 V/AT	[5,26]
Graphene/SiC	3000	83 V/AT	[27,28]
*III–V semiconductors (2D)*			
GaAs/AlGaAs	7000	1250 V/AT	[29,30,31]
AlGaN/GaN	1500	62.5 V/AT	[32,33]
InAs/AlSb	22,000	625 V/AT	[2,31,34]
InSb QW	42,000	1250 V/AT	[7,35,36]
*III–V semiconductors (3D)*			
InSb MBE	35,000	3.1 × $10^{-4}$ m^3^/C	[36,37,38,39]
InAs film	22,000	4.2 × $10^{-5}$ m^3^/C	[2,31,34]
*Other platforms*			
Si CMOS	1400	6.3 × $10^{-4}$ m^3^/C	[10,31]
Bismuth	15,000	2.1 × $10^{-5}$ m^3^/C ^*a*^	[40]
Bi_2_Se_3_ TI	3000	312 V/AT	[11,41]

^*a*^ Single-band value 1/(ne) for the preset carrier density ($n=3\times 10^{23}$ m^−3^); the Hall constant measured on compensated bismuth films is far smaller (of order 10^−7^ m^3^ C^−1^ at room temperature [40]), so this entry is an upper bound on $R_{H}$.

**Table 2:** Bead library. ϕ: magnetic (iron oxide) volume fraction, computed from the iron content (mg/g) reported by Fonnum et al. [18] using $\rho_{\gamma {\mathrm{-Fe}}_{2}O_{3}}=4.9\;g/cm^{3}$. $\mu_{r}$: relative permeability of the ferrite core.

Bead Preset	$d_{b}$ (μm)	ϕ (%)	$\mu_{r}$	Source
Dynabeads MyOne	1.05	13 ^*a*^	10	[18]
Dynabeads M-280	2.83	5 ^*a*^	10	[18]
Dynabeads M-450	4.50	9 ^*a*^	10	[18]
Magnetite 2 μm	2.00	100	10	[12]
Magnetite 6 μm	6.00	100	10	[12]
Magnetite 10 μm	10.0	100	10	[12]

^*a*^ Volume fraction computed from Fonnum et al.’s iron content: MyOne 255 mg/g, M-280 118 mg/g, M-450 202 mg/g; converted via Fe_2_O_3_ stoichiometry and bead density.

### 2.7. Browser Implementation

**Figure 1:**
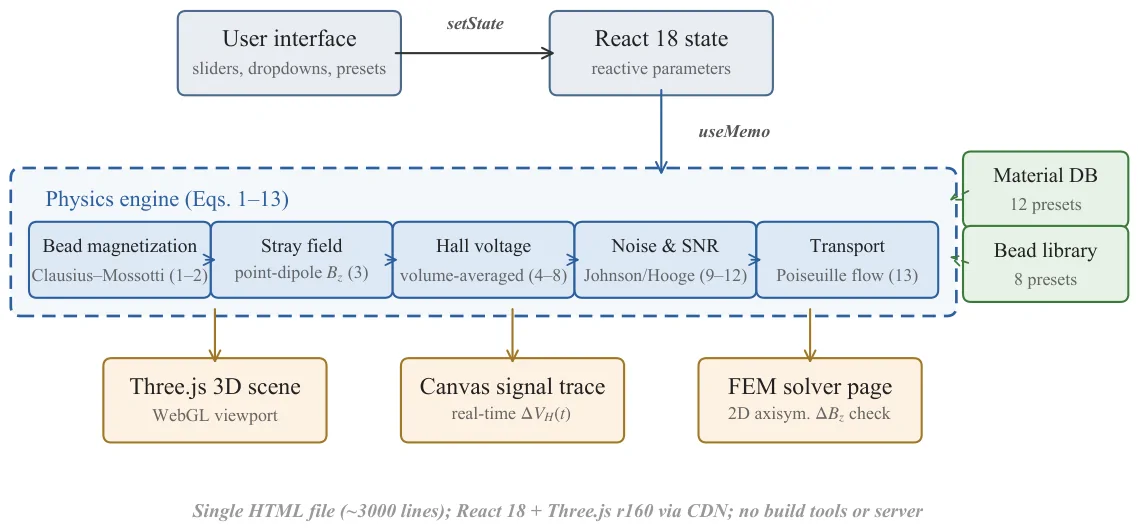
Data-flow architecture of the browser implementation. User interactions update React state, which triggers memoized physics computations (Equations (1)–(13): magnetization, stray field, volume-averaged Hall voltage, noise, and transport). Results feed the Three.js 3D scene, the HTML5 Canvas signal trace, and the 2D axisymmetric FEM solver. The simulation loop uses adaptive sub-stepping to capture narrow signal peaks; parametric sweeps bypass the animation loop and evaluate the equations directly.

The model is provided as a freely accessible browser implementation that evaluates Equations (1)–(13) in a web browser. The application is a single HTML file (∼3000 lines) that loads React 18 (UMD build) for reactive state management and Three.js r160 (ES module) for the WebGL 3D scene from CDN URLs; an HTML5 Canvas renders the 2D signal trace, the parametric sweep charts, and the FEM field visualization. The architecture requires no build tools, package managers, or server-side dependencies, and the same file can be opened from a local filesystem. A pure-JavaScript physics engine exposes the bead-moment, point-dipole, volume-average, and flow-velocity functions plus a helper that derives the Hall coefficient, conductivity, and sensitivity from material parameters and an optional gate voltage. Figure 1 shows the data-flow architecture: user interactions update React state, which triggers the memoized physics computations, whose results feed the Three.js scene, the Canvas trace, and the FEM solver.

**Figure 2:**
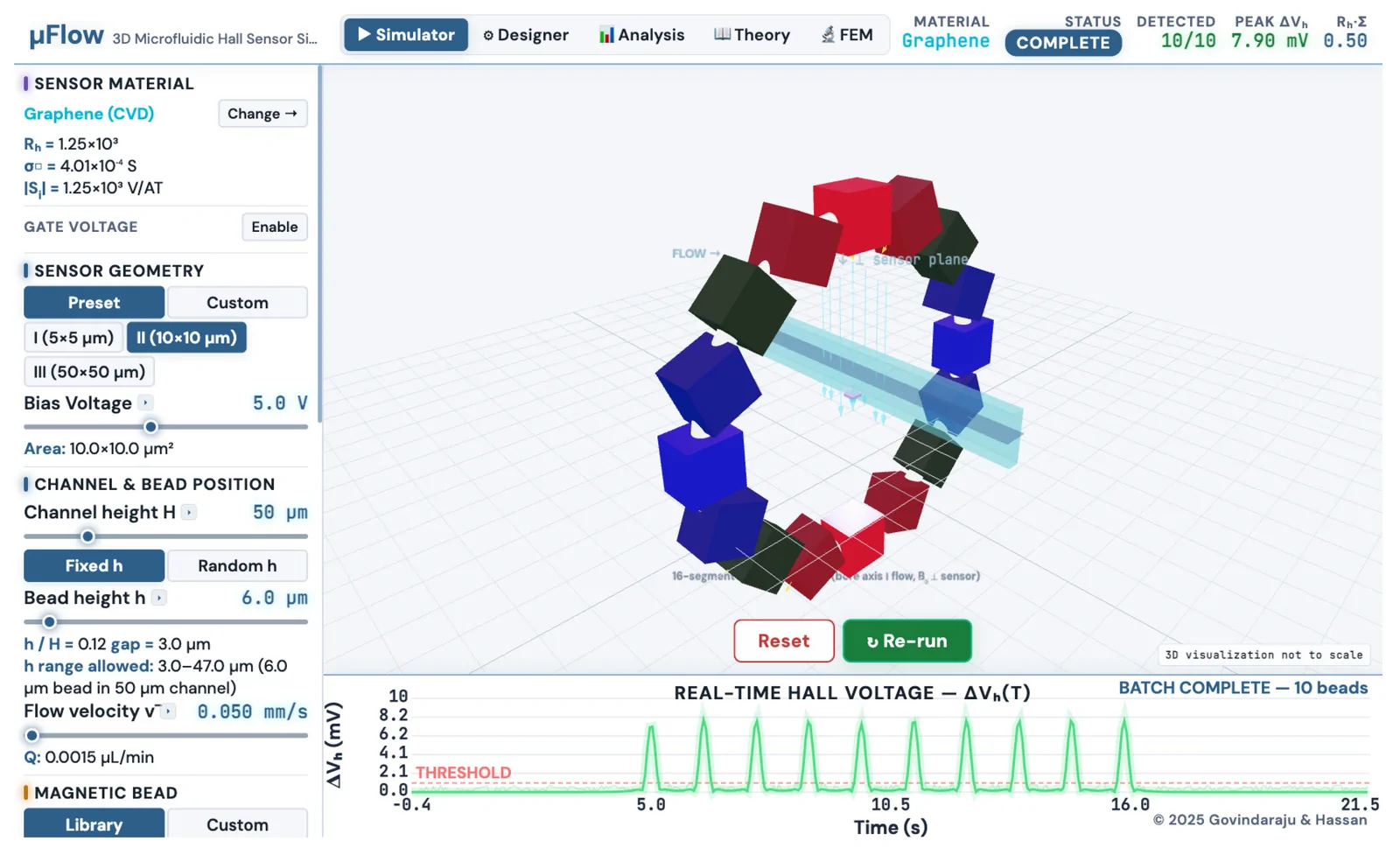
Simulator page at the end of a ten-bead batch at the Design II geometry (w=10 μm, t=1 μm, CVD-graphene preset ($\sigma_{□}=4.0\times 10^{-4}\;S$), magnetite 6 μm bead at fixed height h=6 μm, $B_{0}=50\;mT$, $V_{\mathrm{bias}}=5\;V$, $\bar{v}=0.05\;mm\;s^{-1}$, the slow-flow imaging regime). The left sidebar exposes material, geometry, channel, and bead controls; the center viewport renders the PDMS channel, the Hall chip, and the 16-segment Halbach array (the ten beads have exited the field of view at batch completion); the blocks are colored by the vertical component of their magnetization (red: downward; blue: upward; dark gray: purely in-plane), with yellow arrows marking each block’s magnetization; the bottom panel plots the recorded $\Delta V_{H}$ trace with a noise-injected overlay and detection threshold (ten of ten beads detected).

The interface is organized into pages reached from a tabbed header: a landing page; a Simulator with real-time bead animation, a live $\Delta V_{H}$ signal trace with superimposed noise, and a batch mode with per-bead detection statistics; a Designer for sensor material, geometry, and bead selection with computed physics properties; an Analysis page hosting the parametric sweep engine with comma-separated values (CSV) export; a Theory page presenting the governing equations and data tables; and a FEM Solver page. Figure 2 shows the Simulator page at the Design II sensor geometry. The benchmark and sweep results reported here use a 16×16×8 volume-averaging grid (Equation (4)), shown to be converged by a study at Design II parameters (Section 3.1); the interactive simulator evaluates the quadrature on a coarser grid to sustain real-time frame rates. The parametric sweep engine evaluates $\Delta V_{H}$ across user-specified ranges of bead diameter, sensor size, flow velocity, applied field, channel height, height above the sensor, or gate voltage by computing the equations directly; in multi-material mode, it iterates over all 12 presets at each sweep point, producing overlaid curves.

#### In-App 2D Axisymmetric FEM Solver

The implementation includes a 2D axisymmetric finite-element magnetostatic solver that cross-checks the point-dipole approximation without external software. The solver computes the perturbation field $\Delta B_{z}$ from a magnetized bead using the magnetic scalar potential formulation, $\frac{1}{r}\partial_{r}\left(r\mu_{r}\partial_{r}\varphi \right)+\partial_{z}\left(\mu_{r}\partial_{z}\varphi \right)=0$, on a structured triangular mesh with adaptive refinement near the bead and sensor, solved by a preconditioned conjugate-gradient method. Four mesh resolutions are available, from coarse (∼3200 elements) to ultra (∼39,200 elements). The FEM page displays the $\Delta B_{z}$ field map, the mesh overlay, and a side-by-side comparison of the FEM and analytical dipole profiles at the sensor plane, with the height-to-radius ratio $h/r_{b}$ reported alongside the result. The size of the reported deviation for the benchmark geometries is given in Section 3.1; the solver lets a user judge where the dipole approximation degrades for any on-axis configuration without a commercial FEM license.

The scope of this solver is deliberately narrow: it addresses the magnetostatic step of the chain only. It cross-checks the point-dipole stray-field approximation against a finite-bead-volume solution for an axisymmetric bead-above-disk configuration, and nothing else. It does not model the Hall-current distribution, the square sensor footprint, laterally offset or transiting beads, hydrodynamic transport, or noise. Those parts of the signal chain are benchmarked instead by the companion 3D FEM study (Section 3.1), which couples magnetostatics to Hall transport, and by the comparisons with published experiments (Section 3.2). The three checks form a hierarchy: full 3D coupled benchmark, in-app magnetostatic cross-check, and experimental order-of-magnitude anchor.

## 3. Results

### 3.1. Benchmark Against the Companion 3D FEM Study

**Figure 3:**
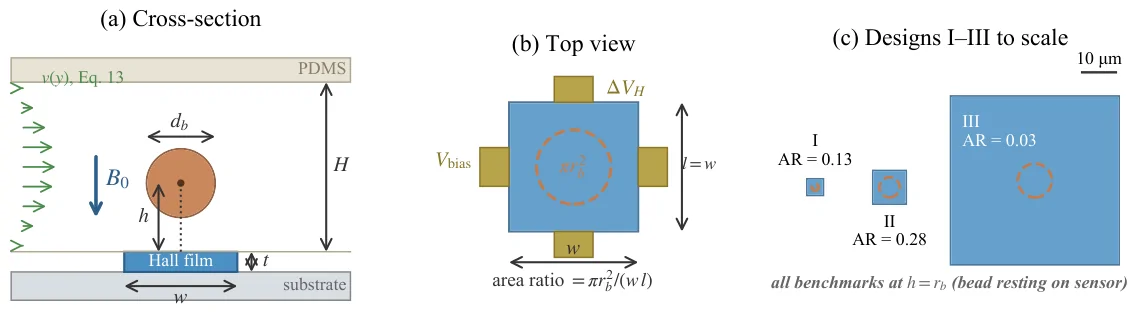
Structural diagram of the model geometry, keyed to Table 3. (**a**) Channel cross-section: sensor width *w* and film thickness *t* of the Hall element, bead diameter $d_{b}$, bead-center height *h* above the sensor surface, channel height *H*, applied field $B_{0}$, and the Poiseuille velocity profile v(y) (Equation (13)). (**b**) Top view: square active area w×l (l=w) with bias contacts and Hall voltage taps; the dashed circle is the bead footprint $\pi r_{b}^{2}$ entering the area ratio $\pi r_{b}^{2}/\left(w\;l\right)$. (**c**) The three benchmark designs drawn to a shared scale; all three sit at $h=r_{b}$, i.e., the bead resting on the sensor surface.

**Figure 4:**
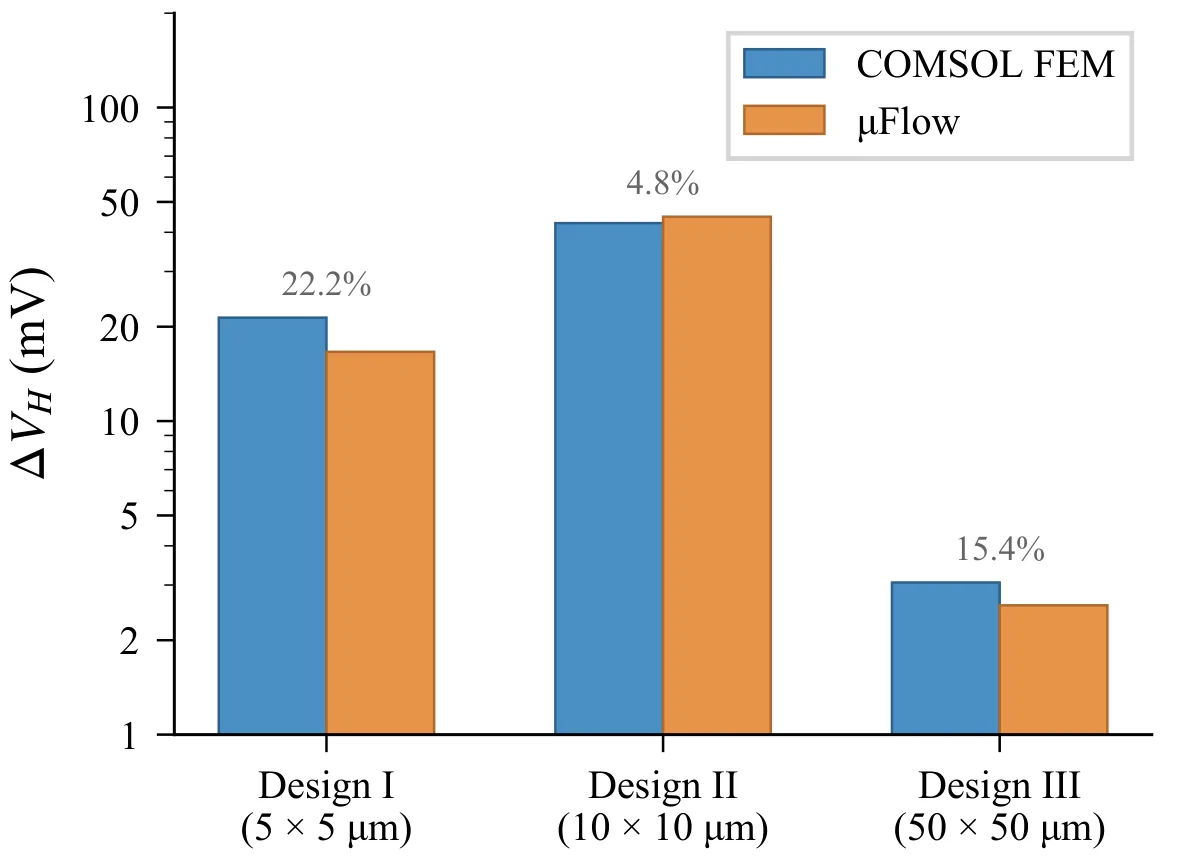
Hall voltage predicted by the analytical–numerical framework versus COMSOL FEM [12] for the three sensor designs; deviation percentages annotated, area ratios as in Table 4.

We benchmark the analytical–numerical framework against the three sensor designs characterized in our companion COMSOL FEM study [12]. The companion model solves the full 3D problem in two coupled stationary steps: a magnetostatic solve with the Halbach source and a nonlinear bead magnetization curve calibrated against published magnetite data, followed by a Hall transport solve in which the magnetostatic field enters an anisotropic conductivity tensor and the Hall voltage is read from floating-potential sense contacts. The benchmark therefore retains exactly the physics that the analytical–numerical chain approximates away: finite bead volume, field non-uniformity over the sensor, and current redistribution. Its outputs were checked internally through non-magnetic control beads (which yield flat traces), linear bias voltage scaling, and a distance decay consistent with a dipolar source [12], and COMSOL modeling of magnetic-particle sensing has also been applied in direct combination with experiment [42]. We use the companion study as an independent, peer-reviewed numerical benchmark rather than as ground truth; the absolute experimental anchor is provided in Section 3.2. Table 3 lists the design parameters, Figure 3 maps each parameter onto the device geometry, and Table 4 presents the benchmark results, also shown graphically in Figure 4.

The analytical–numerical framework reproduces the COMSOL Hall voltage to within 4.8% at the favorable bead-to-sensor area ratio of Design II. At the off-optimum Designs I and III, the point-dipole approximation deviates by 22% and 15%, consistent with its known near-field limit at $h/r_{b}=1$, the regime in which the multipole expansion underlying the dipole form loses accuracy [14]. All three designs operate at $h/r_{b}=1$, so the design-to-design spread is not an $h/r_{b}$ effect alone but a function of the bead-to-sensor area ratio. Design II’s area ratio of 0.28 concentrates the bead’s stray-field footprint within the sensor active area, where the dipole approximation captures most of the volume-averaged field. Design I (area ratio 0.13) under-covers the bead’s near field with a small sensor, so the dipole underestimates the actual field by missing the finite-volume contribution. Design III (area ratio 0.03) over-averages the bead’s field across a large sensor, so the residual near-field error has a larger fractional impact on the small mean. This pattern is the expected behavior of the multipole expansion near the bead surface [14] and motivates the in-app FEM solver (Section In-App 2D Axisymmetric FEM Solver) for users working at $h/r_{b}<1.5$, where the dipole approximation degrades.

The volume-averaging quadrature is converged at the resolution used. At Design II parameters, the 16×16×8 grid gives $\Delta V_{H}=44.86\;mV$, within 0.044% of a 64×64×32 reference; the coarser 8×8×4 grid deviates by 0.18% and the finer 32×32×16 by 0.009%. The quadrature error is therefore negligible compared with the point-dipole near-field error. The in-app FEM solver provides an independent cross-check: for the bead-comparable Designs I and II it reports dipole-model deviations of order ten to twenty percent, consistent with the finite-bead volumetric effects the dipole approximation neglects; for the large Design III sensor, whose disk-averaged dipole field is a small residual of canceling contributions from the positive and negative lobes of $B_{z}$, the relative deviation is of order 100% and is not a meaningful accuracy measure. The two comparisons probe different quantities and are therefore not expected to coincide: the in-app solver compares only the axisymmetric magnetostatic field average, while the COMSOL benchmark includes Hall-current redistribution and the square sensor footprint.

### 3.2. Comparison with Published Detection Experiments

To assess the complete signal chain beyond the COMSOL comparison, we check the model against published microfluidic Hall-effect bead-detection experiments. Exact quantitative reproduction is limited because the published reports do not always specify the precise bead–sensor distance, bias conditions, and sensor geometry needed to reconstruct the measurement; we therefore assess consistency at the order-of-magnitude level.

Mihajlović et al. detected a single commercial 1.2 μm bead with an InAs quantum-well micro-Hall sensor at room temperature, observing a ∼2 μV Hall voltage drop corresponding to a sensed stray-field change of ∼80 μT [2]. The bead signal is therefore of the order of a microvolt. The model’s InAs/AlSb two-dimensional electron gas (2DEG) preset ($S_{I}=625\;V\;A^{-1}\;T^{-1}$) predicts $\Delta V_{H}\approx 1\;\mu V$ for a micron-scale configuration (a 2.8 μm bead with ϕ=0.03 at h=2 μm over a 5 μm sensor), and the characteristic bipolar waveform arises from the sign change in $B_{z}$ at lateral offsets (Equation (3)). The predicted signal magnitude agrees with the experimental value to within a factor of two.

Besse et al. detected a single M-280 bead with a 2.4×2.4 μm Si CMOS Hall sensor ($S_{I}=175\;V\;A^{-1}\;T^{-1}$, R=8.5 kΩ) at $I_{\mathrm{bias}}=0.3\;mA$, with the bead at h=7 μm and the sensor thermal-noise-limited magnetic field resolution at $∼0.2\;\mu T/\sqrt{\mathrm{Hz}}$; they measured magnetic induction variations exceeding 20 μT [10]. The corresponding measured Hall voltage, $S_{I}I_{\mathrm{bias}}\Delta B\approx 1\;\mu V$ for the 20 μT induction change, is in the microvolt range. The model reproduces the order of the bead stray-field magnitude (∼10–100 μT for an M-280 at micron distances) but its signal chain output for the Si CMOS preset is sensitive to the bias and film thickness values, which the report underspecifies; we therefore claim agreement only at the order-of-magnitude level for the magnetic induction.

Table 5 collects the reported conditions and signals of four single-bead Hall detection experiments on three sensor platforms (InAs, Si CMOS, and InSb, the last represented by two experiments [38,39]). The reported noise floors (∼0.08–0.5 μT/$\sqrt{\mathrm{Hz}}$, at bias currents between 50 μA and 1 mA) are the experimental anchor for the noise framework of Section 2.4.

For the intended use—design-stage screening and ranking of geometries and materials before fabrication—this level of agreement is sufficient: the quantities that drive design decisions are rankings and relative comparisons across geometries and materials. The benchmark confirms the framework reproduces the design ranking and the absolute signals to 4.8–22%, and cross-material relations follow directly from the drive-constraint algebra of Section 3.3, while absolute signals in published experiments depend on parameters the reports do not fully specify (exact bead–sensor distance, bead-to-bead moment dispersion, and drive and readout details). Two structural differences between the model and the experiments deserve emphasis. First, most experiments detect surface-bound or resting beads with AC-susceptibility lock-in readout, whereas the model computes the DC transit signal of a flowing bead; the AC scheme shifts detection above the 1/f corner and improves the noise floor relative to the DC estimate. Second, the experimental beads are polymer-coated composites (ϕ<1, Table 2), whereas the FEM benchmark beads are pure magnetite; the volume fraction correction of Equation (1) bridges the two. Absolute prediction of a specific experiment therefore requires the device-specific parameters, and the model’s role is to bound the expected signal and rank the candidates.

We do not include the Gabureac et al. nanocomposite sensor in the quantitative comparison set: its sub-200 nm active area is much smaller than the bead, so the stray field extends well beyond the sensor boundaries, a geometry that falls outside the model’s validated regime [4].

### 3.3. Cross-Material Comparison

**Figure 5:**
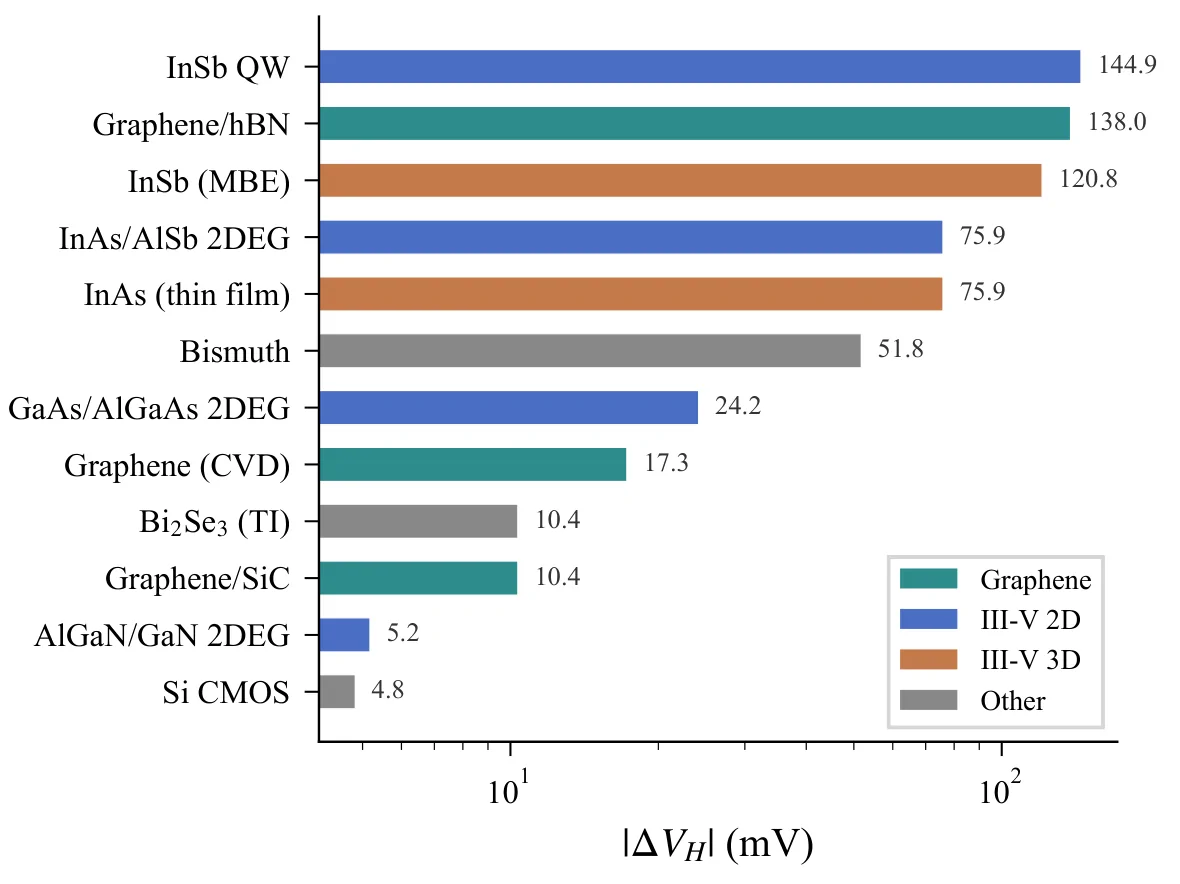
Cross-material comparison of $\Delta V_{H}$ under identical geometry and bead conditions at constant-voltage drive (Design II, $B_{0}=50\;mT$, $V_{\mathrm{bias}}=5\;V$ for every material), bars color-coded by material family. Under this constraint, the signal tracks carrier mobility: InSb quantum wells and graphene/hBN produce the strongest signals, while Si CMOS and AlGaN/GaN are among the weakest. Table 7 reports how the ranking changes under constant-current and constant-power drive.

Figure 5 compares the Hall voltage produced by all 12 material presets under identical Design II geometry and bead conditions (6 μm magnetite bead, h=3 μm, $B_{0}=50\;mT$, $V_{\mathrm{bias}}=5\;V$). Table 6 then adds the noise-aware metrics for a representative subset, and Table 7 reports how the ranking changes when the electrical drive constraint is changed.

The ranking depends explicitly on the electrical drive constraint, and the two natural constraints rank by different material properties. At constant bias voltage (Figure 5), $\Delta V_{H}=G\;\mu \;\langle B_{z}\rangle V_{\mathrm{bias}}$, so the mobility ratio between two materials predicts their signal ratio: InSb quantum wells (μ = 42,000 cm^2^ V^−1^ s^−1^) and graphene on hexagonal boron nitride (hBN) (μ = 40,000 cm^2^ V^−1^ s^−1^) produce the strongest signals, while Si CMOS ($\mu =1400\;cm^{2}\;V^{-1}\;s^{-1}$) and AlGaN/GaN ($\mu =1500\;cm^{2}\;V^{-1}\;s^{-1}$) are among the weakest. At constant bias current, by contrast, $\Delta V_{H}=G\;S_{I}I_{\mathrm{bias}}\langle B_{z}\rangle \propto 1/n_{s,\mathrm{tot}}$ ranks materials by total sheet carrier density and is independent of mobility; this is why InSb QW, CVD graphene, and Si CMOS, whose total sheet densities coincide at $5\times 10^{15}$ $m^{-2}$, show identical $\Delta V_{H}=0.086\;mV$ in Table 6. This signal ranking alone is insufficient for sensor selection because it ignores the material-specific noise floor. Table 6 reports the noise-aware metrics for a representative subset at Design II geometry under constant-current drive (w=10 μm, $I_{\mathrm{bias}}=10\;\mu A$, $\Delta f=f_{\mathrm{op}}=1\;k\mathrm{Hz}$, T=300 K). At these conditions, graphene/hBN ($\Delta V_{H}=0.39\;mV$, $\alpha_{H}=10^{-5}$) produces about 4.5× the signal of Si CMOS ($\Delta V_{H}=0.086\;mV$, $\alpha_{H}=5\times 10^{-3}$), and its higher SNR is reinforced by the lower Hooge parameter; the full 12-material set spans a wider range. Because $V_{1/f}\propto \sqrt{\alpha_{H}/N}$ with $N=n_{\mathrm{tot}}A$, a platform with low carrier density (high $S_{I}$) can suffer higher 1/f noise from a reduced carrier count, so the SNR ranking does not always follow the $\Delta V_{H}$ ranking. The Designer page of the browser implementation reports $V_{n}$, SNR, and $B_{\min}$ for any user-specified $\alpha_{H}$, and the ridge analysis of Section 3.4 quantifies this trade-off across sensor width.

Table 7 reports the comparison under all three constraints, computed from the same material database and noise framework. The signal ranking inverts between constant voltage (mobility-ranked, InSb QW first) and constant current (density-ranked, graphene/hBN first), while the SNR ordering is stable across constraints because, in the 1/f-dominated regime, the SNR reduces to $\mu \sqrt{n_{s,\mathrm{tot}}/\alpha_{H}}$ times a geometric factor, a combination that is independent of the drive; $\alpha_{H}$ alone sets only the extremes (graphene/hBN’s lead, Si CMOS’s floor). $B_{\min}$ improves with drive level while a platform remains thermally limited (graphene/hBN: 106 to 64 nT/$\sqrt{\mathrm{Hz}}$ between 10 μA and 0.1 V, as it crosses into the 1/f-dominated regime) and is essentially drive-independent for platforms already 1/f-dominated, such as Si CMOS (∼19 μT/$\sqrt{\mathrm{Hz}}$ under all three constraints). No comparison in this paper mixes constraints; each figure and table caption states its drive condition.

CVD graphene on SiO_2_ ($\mu =5000\;cm^{2}\;V^{-1}\;s^{-1}$) offers a practical intermediate: its signal is well above Si CMOS while CVD growth is compatible with wafer-scale integration [3]. AlGaN/GaN produces signals comparable to Si CMOS but operates reliably above 500 K [32], and graphene/SiC is stable to 770 K [27], so both remain viable where graphene and InSb would degrade.

### 3.4. Geometry and Sensitivity Map

**Figure 6:**
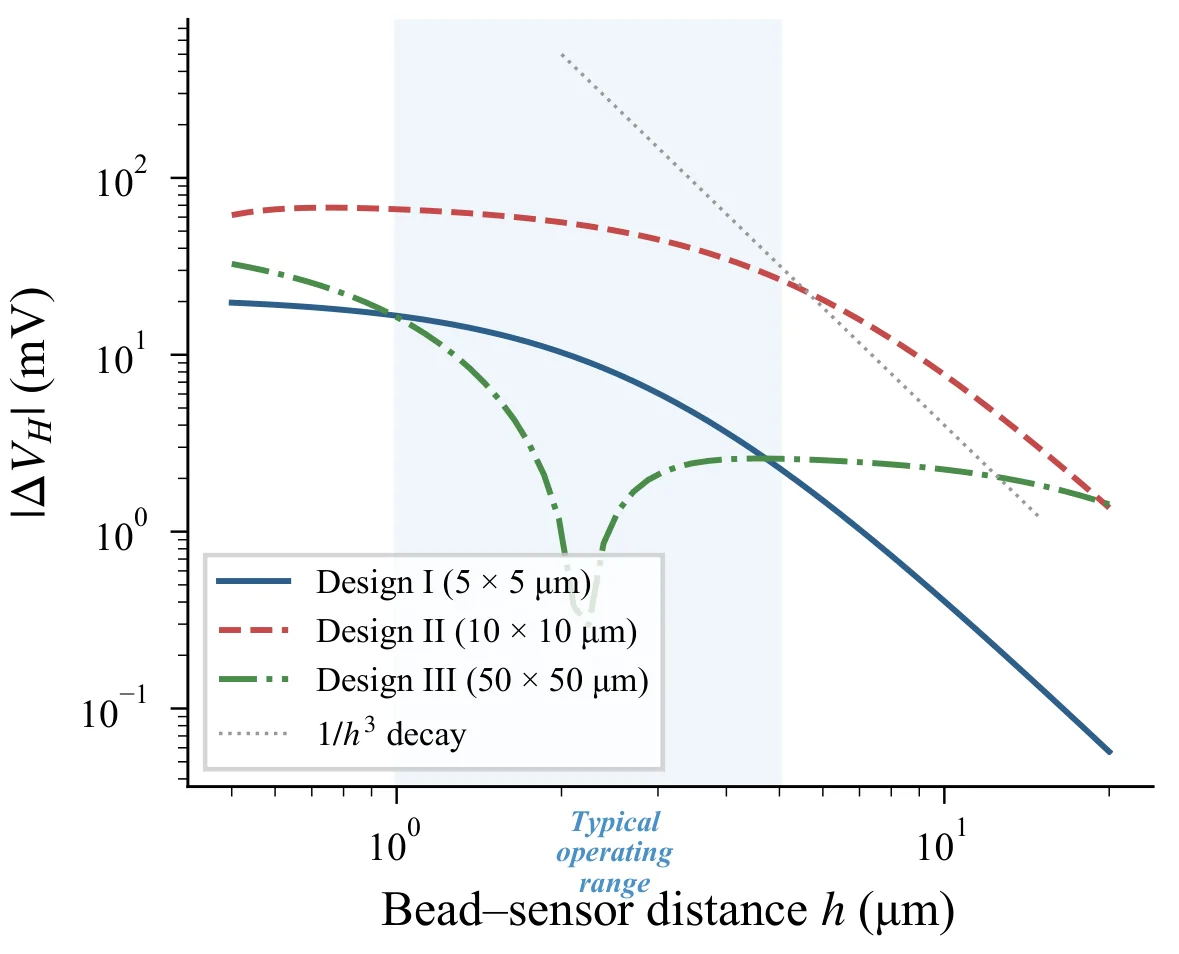
Hall voltage $\Delta V_{H}$ versus bead–sensor distance for the three benchmark sensor designs, each with its own bead (Table 3). The $1/h^{3}$ dipolar decay is evident on the log-log scale for Designs I and II. Design II provides the strongest signal within the typical microfluidic operating range (h= 1–5 μm), reflecting its larger bead moment ($m\propto r_{b}^{3}$) relative to its sensor dilution; the cusp in the Design III curve is the zero-crossing of the volume-averaged $\Delta B_{z}$.

**Figure 7:**
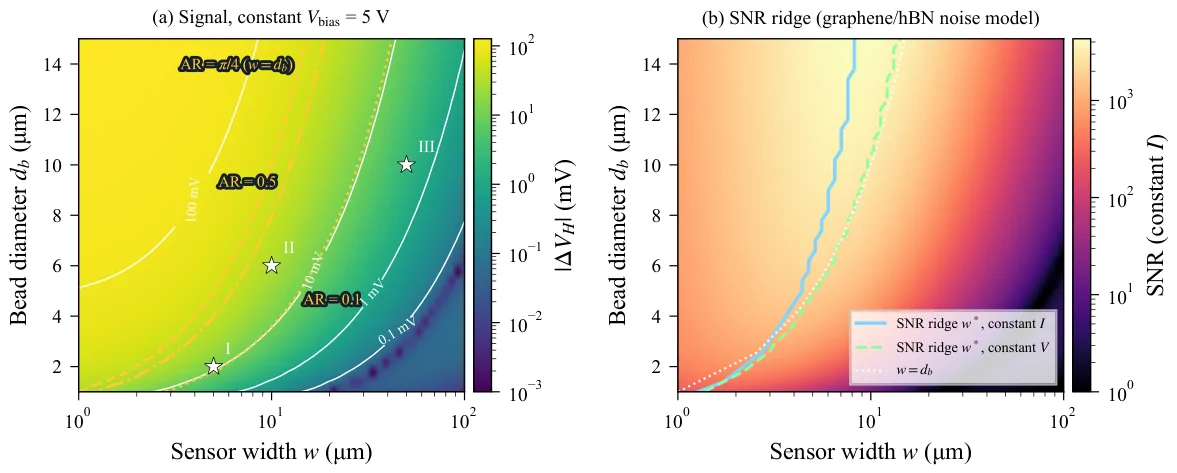
Controlled sensitivity map at fixed $h/r_{b}=1.5$ ($B_{0}=50\;mT$, t=1 μm, magnetite beads, 16×16×8 quadrature). (**a**) $|\Delta V_{H}|$ at constant $V_{\mathrm{bias}}=5\;V$ with iso-signal contours (white), area ratio iso-lines $\pi r_{b}^{2}/\left(wl\right)=0.1$, 0.5, and π/4 (equivalent to $w=d_{b}$) in gold, and the three benchmark designs as stars (geometry markers only: the map is evaluated at $h/r_{b}=1.5$, whereas the benchmarks sit at $h/r_{b}=1$); the signal-only optimum sits at the smallest sensor width, with no interior ridge. (**b**) SNR map under the constant-current noise conditions of Table 6 (graphene/hBN), with the extracted SNR ridge $w^{*}\left(d_{b}\right)$ under constant-current (solid) and constant-voltage (dashed) drive; both lie within a factor of two of the bead-comparable line $w=d_{b}$ (dotted), with the constant-voltage ridge tracking it closely.

Figure 6 shows $\Delta V_{H}$ versus bead–sensor distance *h* for the three sensor sizes, computed by sweeping *h* over 0.5–20 μm. Designs I and II approach the far-field $1/h^{3}$ dipolar decay within the plotted range; the large Design III sensor remains in its averaging-dominated regime. Because each design carries its own bead, the curves confound bead and sensor size: Design II is the strongest over nearly the entire sweep (Design III overtakes it only at the largest plotted distances), since its 6 μm bead carries ∼27× the moment of Design I’s 2 μm bead ($m\propto r_{b}^{3}$). The cusp in the Design III curve marks the zero-crossing of the volume-averaged $\Delta B_{z}$ over the large sensor. At fixed bead size, smaller sensors produce stronger per-bead signals but are more sensitive to lateral misalignment, while larger sensors tolerate bead-position uncertainty at the cost of geometric dilution; the controlled sweep of Figure 7 separates these effects.

Figure 7 charts the bead diameter versus sensor width plane in a controlled sweep: both panels are computed at fixed $h/r_{b}=1.5$, inside the dipole model’s regime of validity, so that bead height does not vary implicitly with bead size. The signal and SNR optima must be distinguished, because they behave differently. The signal alone (Figure 7a) has no interior optimum in sensor width: $|\Delta V_{H}|$ increases monotonically as the sensor shrinks toward the bead footprint, because a smaller active area concentrates the volume-averaged field, so the voltage-only optimum sits at the smallest sensor that the fabrication process allows, and the diagonal structure in the map traces iso-signal contours rather than a ridge. An interior optimum appears when noise is included (Figure 7b). For a square sensor, the thermal noise is width-independent ($R_{□}$ is a sheet property) while the Hooge 1/f noise scales as 1/w through the carrier count $N=n_{s}w^{2}$ (Equation (11)), so shrinking the sensor eventually costs more in 1/f noise than it gains in field concentration. Extracting the SNR ridge $w^{*}\left(d_{b}\right)=\arg\max_{w}\mathrm{SNR}$ yields a bead-tracking optimum: under the constant-current conditions of Table 6 (graphene/hBN), $w^{*}/d_{b}=0.55$–1.26 (median 0.72) across $d_{b}=$ 1–15 μm, corresponding to bead-to-sensor area ratios of 0.5–2.6; under constant-voltage drive, where the ridge is material-independent in the 1/f-dominated regime that holds for all presets at this drive, $w^{*}/d_{b}=0.90$–1.37 (median 0.99), corresponding to area ratios of 0.4–1.0. The design rule is therefore that the SNR-optimal sensor width is comparable to the bead diameter, $w^{*}\approx d_{b}$. For Dynabeads M-280 ($d_{b}=2.83\;\mu m$), the extracted ridge gives $w^{*}\approx$ 2.9–3.2 μm across the two drives; for M-450 ($d_{b}=4.5\;\mu m$), $w^{*}\approx$ 3.9–4.7 μm. This is consistent with the geometries of published single-bead experiments, in which the Hall cross is comparable in size to the bead: Mihajlović et al. used a ∼1 μm cross for a 1.2 μm bead ($w/d_{b}\approx 0.8$) [2,17]. Accuracy, the third consideration the sweep controls for, has no interior optimum in this plane: it enters as the regime constraint on $h/r_{b}$, held at 1.5 here, with the dipole error at bead-comparable geometries of order ten to twenty percent as reported by the in-app solver (Section 3.1), and with the residual area-ratio dependence of the benchmark deviations (Table 4) analyzed in Section 3.1 and recorded among the limitations (Section 4.3).

### 3.5. Flow-Velocity Sensitivity

**Figure 8:**
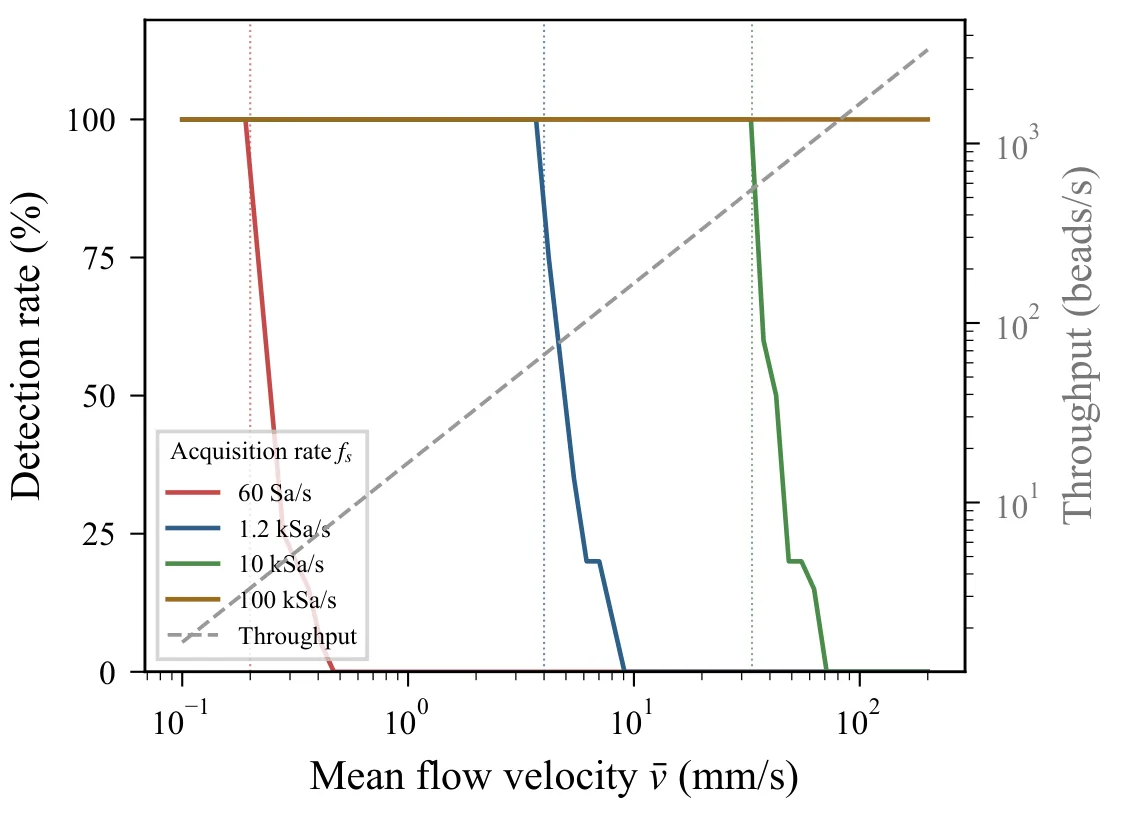
Sampling-limited flow-velocity window. Detection rate versus mean flow velocity for acquisition rates $f_{s}=60$, $1.2\times 10^{3}$, $10^{4}$, and $10^{5}$ Sa/s (Design II, H=25 μm, N=20 beads at uniform random heights with a fixed seed, threshold 10 μV, $N_{\min}=2$ samples per transit, single-sample detection with no additional integration time). Dotted vertical lines mark the closed-form cutoff of Equation (14) (the $10^{5}$ Sa/s cutoff, 333 mm $s^{-1}$, lies beyond the plotted range); the gray dashed curve is the throughput at the simulator’s bead seeding spacing of 6w. The detection cliff scales linearly with $f_{s}$; the lower edge of a practical operating window is a throughput choice, not a detection limit.

The upper limit of the usable flow velocity is set by the readout sampling, not by the signal chain physics: the peak $\Delta V_{H}$ of a transiting bead depends only on its height and the geometry, so the question is whether the acquisition captures the peak during the transit. We therefore model the readout explicitly as a sampled acquisition at rate $f_{s}$ and count a bead as detected when (i) its peak $|\Delta V_{H}|$ exceeds the threshold and (ii) at least $N_{\min}$ samples fall within its transit, $f_{s}\;w/v_{\mathrm{local}}\geq N_{\min}$. For a bead near the channel midplane ($v_{\mathrm{local}}=1.5\bar{v}$), the second condition gives a closed-form upper bound,

(14) $${\bar{v}}_{\max}\approx \frac{wf_{s}}{1.5\;N_{\min}},$$

which scales linearly with the acquisition rate. Figure 8 shows the detection rate versus $\bar{v}$ for a family of acquisition rates with all readout settings stated: Design II geometry, channel height H=25 μm, N=20 beads at uniform random heights (fixed seed), threshold 10 μV (lock-in class; the threshold never binds for this geometry, where the minimum peak is 1.1 mV), $N_{\min}=2$ samples per transit, and no additional integration time (single-sample detection). For w=10 μm, Equation (14) gives ${\bar{v}}_{\max}\approx 0.2\;mm\;s^{-1}$ at $f_{s}=60\;\mathrm{Hz}$, 4 mm $s^{-1}$ at 1.2 kHz, and 33 mm $s^{-1}$ at 10 kHz; sub-kilohertz lock-in detection is established practice in single-bead Hall experiments [10], graphene Hall noise has been characterized into the kilohertz range [43], and integrated CMOS Hall arrays parallelize readout across $10^{4}$ pixels [44]. A flow-velocity window of 0.5–5 mm $s^{-1}$ therefore corresponds to a ∼1 kHz-class acquisition; it is a property of the readout, and faster acquisition extends it proportionally.

At low velocities, detection is unconditional and the trade-off is throughput, which grows linearly with $\bar{v}$ (dashed curve); the lower edge of a practical window is therefore a throughput choice rather than a detection limit. Within the window, the peak $\Delta V_{H}$ is governed by bead height, not velocity. This behavior is consistent with the slow-flow regime of Aledealat et al., who imaged beads transiting the Hall cross at $∼3\;\mu m\;s^{-1}$ (1 μL $\min^{-1}$), giving second-scale transits that resolved the bipolar Hall transient as each bead entered, covered, and left the cross [45]. A full treatment of the noise–bandwidth trade-off (longer integration lowers the noise floor but must fit inside the transit) requires the frequency-resolved noise data of the specific readout chain [43] and is outside the scope of the present model.

### 3.6. Batch Detection Statistics

Batch mode injects *N* beads (1–50) and records per-bead peak $\Delta V_{H}$, bead height, and detection status. Table 8 reports the output at the Design II sensor geometry with the CVD-graphene preset, $B_{0}=50\;mT$, and $V_{\mathrm{bias}}=5\;V$ (the material and field settings of Figure 2) for 10 beads at fixed height (h=3.0 μm, the bead resting on the sensor) and in range mode (*h* drawn uniformly from 3–10 μm; the lower bound is the bead radius, below which the bead would intersect the sensor plane).

The fixed-height mean is consistent with the benchmark: the 44.86 mV of Table 4 scales to 17.3 mV at the CVD-graphene mobility ($R_{H}\sigma =\mu =0.5\;m^{2}\;V^{-1}\;s^{-1}$ rather than the benchmark $1.3\;m^{2}\;V^{-1}\;s^{-1}$), and the residual 2% is the mean shortfall from the two sampling effects described next, both of which can only lower the sampled peak. In fixed-height mode, the low coefficient of variation (1.7%) has two comparable sources: the random lateral offset (within ±0.2w) that the simulator assigns to each trajectory, and the finite sub-step at which the peak is sampled; the physics contributes no spread. In range mode, the coefficient of variation rises to 48.1% because beads at different heights experience different stray-field amplitudes. Detection remains complete over this span: at the top of the range the peak is still about three times the 1 mV threshold, so height dispersion degrades signal uniformity here without yet costing sensitivity. This height-dependent variation is physically realistic: Aledealat et al. recorded bipolar Hall transients from superparamagnetic beads (2.6 μm; Bangs Laboratories, Inc., Fishers, IN, USA) transiting an InAs micro-Hall cross, with transient amplitudes set by the bead’s stray field and its position relative to the sensor, consistent with the $1/h^{3}$ scaling of the volume-averaged field [45].

### 3.7. Computational Efficiency

The analytical–numerical substitution replaces the volumetric FEM solve with a direct evaluation of Equations (1)–(5). Table 9 reports indicative costs. A single $\Delta V_{H}$ evaluation completes in under 1 ms; a 100-point geometry sweep that we estimate at ∼8 h from per-configuration solve times of the companion COMSOL model [12] finishes in 0.3 s. The speedup is a property of the analytical approximation, not an algorithmic improvement over FEM, and applies only in the regime where the model is validated; outside it, the cost advantage is moot and the in-app FEM solver or an external package is the appropriate tool.

## 4. Discussion

### 4.1. Design Rules

The parametric analysis yields three design rules. First, the SNR-optimal sensor width is comparable to the bead diameter, $w^{*}\approx d_{b}$ (Figure 7b), corresponding to bead-to-sensor area ratios of 0.4–1.0 at constant voltage and 0.5–2.6 at constant current; signal alone, by contrast, favors the smallest sensor the process allows, so the bead-comparable rule is a property of the noise trade-off, not of the voltage map. The rule matches the bead-comparable Hall crosses used for single-bead detection in practice [2,17]. Second, the usable flow-velocity window is sampling-limited: detection holds up to ${\bar{v}}_{\max}\approx wf_{s}/\left(1.5N_{\min}\right)$ (Equation (14)), about 4 mm $s^{-1}$ for a 10 μm sensor at a kilohertz-class acquisition rate, and the lower edge of a practical window is a throughput choice. Third, material selection requires noise-aware analysis under an explicit drive constraint: constant-voltage drive ranks signals by mobility while constant-current drive ranks them by carrier density (Table 7), and because the SNR combines mobility, carrier density, and the Hooge parameter (Table 6), a high-mobility platform with poor noise performance can achieve a lower SNR than a lower-mobility platform with superior $\alpha_{H}$.

### 4.2. Regime of Validity of the Dipole Model

The benchmark in Section 3.1 sets quantitative bounds on the point-dipole approximation. Because all three benchmark designs sit at $h/r_{b}=1$, the limit of the dipole expansion, the benchmark deviations (Table 4) represent an upper bound on the model error: predictions are expected to become more reliable away from the near-contact regime and should be treated as approximate near contact. For near-contact geometries ($h\to r_{b}$), relevant to magnetic capture or surface-bound assays, the multipole corrections that only a full FEM solver provides are needed; the in-app 2D axisymmetric solver supplies this check without a commercial license. The bead magnetization model is similarly bounded to $B_{0}≲200\;mT$, above which a Langevin ensemble model is required, and the transport model assumes fully developed laminar flow valid for Re<1 [16].

### 4.3. Limitations

The model’s assumptions bound its applicability, and we state them together here.

*Benchmark coverage:* the FEM benchmark comprises three designs, each at a single bead position directly above the sensor center and all at $h/r_{b}=1$; our comparison uses only these closest-approach peak values, and the deviation depends on the bead-to-sensor area ratio (from 4.8% at an area ratio of 0.28 to 22% at 0.13). The companion study reports its distance sweeps graphically, and digitizing them for off-contact benchmarking is planned; until then the in-app axisymmetric solver is the numerical check away from contact.

*Bead-height distribution:* range mode draws bead heights from a uniform distribution as a deliberately conservative case, and the constant downward drift ($∼0.3\;\mu m\;s^{-1}$) that approximates magnetic capture is an ad hoc value, smaller than the Stokes sedimentation velocity ($∼2\;\mu m\;s^{-1}$ for an M-280); the true height distribution in a device depends on sedimentation, lift, and magnetophoresis, which the model does not resolve.

*Bead–bead coupling and chaining:* the model treats each bead independently. This fails at high concentration or high field, where dipolar coupling assembles beads into chains along the applied field; the underlying dipole–dipole interactions are quantified by superparamagnetic interaction theory [19], and field-induced chaining of magnetic microparticles is strong enough that it is deliberately exploited in microfluidic device fabrication [46]. The contact coupling energy is large: for an M-280 at $B_{0}=50\;mT$ (ϕ=0.05), $\lambda =\mu_{0}m^{2}/\left(2\pi d_{b}^{3}k_{B}T\right)\approx 6\times 10^{3}$, so two touching beads bind irreversibly and the operative control is concentration. Keeping the mean interparticle spacing above five bead diameters requires number densities below $∼3.5\times 10^{5}$ beads per μL for M-280 ($∼7\times 10^{6}$ for MyOne), comfortably above typical single-bead assay concentrations. At that spacing the pair interaction still exceeds $k_{B}T$ ($\lambda /5^{3}\approx 48$), so the dilute regime is protected by kinetics rather than thermodynamics: the magnetophoretic drift velocity of each bead toward its neighbor at five diameters ($∼1.6\;\mu m\;s^{-1}$ for M-280) is far slower than the transit velocity through the sensing zone, so encounters are rare on the assay timescale and the independent-bead treatment holds. Near the magnet, the field gradient additionally drives magnetophoretic accumulation, which raises the local concentration above the bulk value and tightens the dilute-regime bound.

*Biofunctionalization:* antibody or streptavidin coatings affect the signal through two channels the model already parameterizes. The magnetic dilution of the coated, composite bead is captured by the volume fraction ϕ (Equation (1)), whose values in Table 2 are derived from measurements of coated commercial beads [18]. The coating shell additionally increases the minimum bead–sensor separation by its thickness δ; recomputing $\langle B_{z}\rangle$ with the bead raised by δ gives signal reductions below 3% for δ≤100 nm at Design II, while for bead-comparable sensors near contact the $1/h^{3}$ scaling bounds the loss at ∼3δ/h. Functionalized beads have been detected with the same micro-Hall platforms modeled here [47]. Binding-induced changes in hydrodynamic radius alter transit velocity but not the peak signal.

*Fabrication tolerances:* the model evaluates nominal geometries. Film thickness enters $S_{I}\propto 1/t$ for 3D materials, so a thickness tolerance maps directly onto a sensitivity tolerance; lateral misalignment of the bead trajectory from the sensor center reduces the peak $\langle B_{z}\rangle$; and the geometric factor *G* departs from unity for non-square elements. Of these, only the film thickness is a user input; the lateral offset is sampled randomly within ±0.2w in the simulator, and *G* is fixed at unity.

*Readout electronics and thermal effects:* the noise framework covers the sensor’s thermal and 1/f noise but not amplifier noise, bias-source noise, or filter response; the flow-window analysis models acquisition as ideal sampling at $f_{s}$. All material parameters are room-temperature values; temperature drift of μ and $n_{s}$ is not modeled, although bias self-heating itself is small (≲0.2 K at 5 V for the benchmark designs [12]) and the high-temperature platforms (AlGaN/GaN, graphene/SiC) retain operation well above 300 K [27,32].

*Experimental validation:* no new experimental data were generated for this study; the model is benchmarked against the companion FEM study and anchored to published experiments at the order-of-magnitude level (Section 3.2). Fabrication and measurement of the bead-comparable designs recommended here are the natural next step.

## 5. Conclusions

We presented a coupled analytical–numerical framework for the microfluidic Hall-effect magnetic-bead detection signal chain, combining Clausius–Mossotti bead magnetization with a volume-fraction correction, a point-dipole stray field, a volume-averaged Hall voltage with geometric correction, Poiseuille transport, and a Johnson–Nyquist/Hooge 1/f noise framework over a database of 12 literature-derived sensor presets, extensible to any platform whose transport parameters are supplied. Benchmarked against a companion COMSOL FEM study, the model reproduces the Hall voltage to within 4.8% at the favorable bead-to-sensor area ratio and to 15–22% at the off-optimum geometries, all evaluated at the dipole near-field limit $h/r_{b}=1$. The model yields design rules: an SNR-optimal sensor width comparable to the bead diameter ($w^{*}\approx d_{b}$; area ratios 0.4–1.0 at constant voltage and 0.5–2.6 at constant current), consistent with the bead-comparable sensor geometries used in reported single-bead detections; a cross-material comparison resolved by electrical drive constraint, in which constant-voltage signals rank by mobility, constant-current signals rank by carrier density, and the SNR combines mobility, carrier density, and the Hooge parameter; and a sampling-limited flow-velocity window ${\bar{v}}_{\max}\approx wf_{s}/\left(1.5N_{\min}\right)$ set by the acquisition rate. The predicted signal magnitudes are consistent at the order-of-magnitude level with published InAs and Si CMOS detection experiments. We release the model as a freely accessible, no-install browser implementation with an in-app 2D axisymmetric magnetostatic FEM solver that quantifies where the dipole approximation degrades; the application is deployed at https://uflow.studio (accessed on 21 August 2026) and runs in any modern browser without installation, registration, or a license.

## Figures and Tables

**Table 3:** Benchmark configurations. The geometries (*w*, *t*, $d_{b}$, *h*) and the sensor parameters ($R_{H}=1.25\times 10^{-3}\;m^{3}\;C^{-1}$, $\sigma =1040\;S\;m^{-1}$, $V_{\mathrm{bias}}=5\;V$) follow the three designs of Reference [12]; the present evaluation uses solid magnetite beads ($\mu_{r}=10$, ϕ=1) at $B_{0}=50\;mT$. The area ratio is $\pi r_{b}^{2}/\left(wl\right)$ for a square sensor (l=w); see Figure 3. The Design III value evaluates to 0.031; the corresponding value printed in Reference [12] (≈0.01) omits the factor π.

Parameter	Design I	Design II	Design III
Sensor width *w* (μm)	5	10	50
Sensor thickness *t* (μm)	0.5	1	1
Bead diameter $d_{b}$ (μm)	2	6	10
Bead-center height *h* (μm)	1	3	5
Bead-to-sensor area ratio	0.13	0.28	0.03

**Table 4:** Benchmark of the analytical–numerical framework against COMSOL FEM results [12], with the bead positioned directly above the sensor center. Sign: whether the model value lies below or above the COMSOL value.

Design	COMSOL (mV)	Model (mV)	Deviation (%)	Sign	Area Ratio
I (5×5 μm)	21.375	16.63	22.2	below	0.13
II (10×10 μm)	42.79	44.86	4.8	above	0.28
III (50×50 μm)	3.056	2.584	15.4	below	0.03

**Table 5:** Published single-bead Hall detection experiments used as the experimental anchor. Values as reported in the cited papers; “—” indicates the quantity is not reported.

Experiment	Sensor	Bead	Reported Signal	Noise Floor
Mihajlović 2005 [2]	InAs QW, ∼1 μm	1.2 μ m	∼2 μV ( 80 μT)	—
Besse 2002 [10]	Si CMOS, 2.4 μm	M-280 at h=7 μm	>20 μT (∼1 μV)	0.2 μT/$\sqrt{\mathrm{Hz}}$
Sandhu 2004 [38]	InSb film, 4.5 μm	M-270, in contact	μT-scale steps	77 nT/$\sqrt{\mathrm{Hz}}$ at 1 mA
Kazakova 2009 [39]	InSb, 1.5 μm double cross	1.0 μ m	DC cross-difference $\Delta V_{H}$	<0.5 μT/$\sqrt{\mathrm{Hz}}$

**Table 6:** Noise-aware metrics at Design II geometry under constant-current drive (w=10 μm, $I_{\mathrm{bias}}=10\;\mu A$ for every material, so $V_{\mathrm{bias}}=I_{\mathrm{bias}}R_{□}$ varies by material, $\Delta f=f_{\mathrm{op}}=1\;k\mathrm{Hz}$, T=300 K). $V_{n}=\sqrt{V_{\mathrm{th}}^{2}+V_{1/f}^{2}}$; $B_{\min}$ is the noise-equivalent field spectral density, band-averaged over Δf (Equation (12)). The 3D preset (Si CMOS) is evaluated at a film thickness of t=0.5 μm (its representative material thickness, rather than the 1 μm of Design II in Table 3); this thickness sets its total sheet density $n_{s,\mathrm{tot}}=n\;t$.

Material	$\alpha_{H}$	$\Delta V_{H}$ (mV)	$V_{n}$ (nV)	SNR	$B_{\min}$ (nT/$\sqrt{\mathrm{Hz}}$)
Graphene/hBN	$1\times 10^{-5}$	0.39	190	2060	106
InSb QW	$5\times 10^{-4}$	0.086	105	820	266
CVD graphene	$1.8\times 10^{-4}$	0.086	444	194	1124
InAs/AlSb	$3.7\times 10^{-3}$	0.043	159	271	806
Si CMOS	$5\times 10^{-3}$	0.086	7440	11.6	18,800

**Table 7:** Cross-material metrics at Design II geometry under three electrical drive constraints: constant current ($I_{\mathrm{bias}}=10\;\mu A$), constant voltage ($V_{\mathrm{bias}}=0.1\;V$), and constant power (P=10 μW). Same noise parameters as Table 6.

Material	$\Delta V_{H}$ (mV)			SNR		
	const. I	const. V	const. P	const. I	const. V	const. P
Graphene/hBN	0.39	2.76	3.29	2060	3415	3433
InSb QW	0.086	2.90	1.58	820	1101	1100
CVD graphene	0.086	0.35	0.55	194	217	218
InAs/AlSb	0.043	1.52	0.81	271	300	300
Si CMOS	0.086	0.097	0.29	11.6	11.6	11.6

**Table 8:** Batch detection statistics at the Design II sensor geometry (w=10 μm, t=1 μm; magnetite $d_{b}=6\;\mu m$ bead; CVD-graphene preset, $B_{0}=50\;mT$, $V_{\mathrm{bias}}=5\;V$; $\bar{v}=1\;mm\;s^{-1}$, N=10 beads, detection threshold 1 mV), as recorded by the interactive simulator. Range-mode values are one stochastic realization; over six repeat runs the mean peak spanned 5.6–10.4 mV and the coefficient of variation 43–62%, with every bead detected in every run.

Statistic	Fixed Height	Range Mode
Mean peak $\Delta V_{H}$ (mV)	16.9	7.06
Standard deviation (mV)	0.28	3.40
Coefficient of variation (%)	1.7	48.1
Detection rate (%)	100	100

**Table 9:** Indicative computational cost on a 2023 MacBook Pro with an Apple M2 processor (Apple Inc., Cupertino, CA, USA) running Google Chrome (Google LLC, Mountain View, CA, USA). The COMSOL times are the authors’ estimates based on per-configuration solve times of the companion-study model [12], which does not itself report timings.

Task	Analytical–Numerical Framework	COMSOL (est.)
Single $\Delta V_{H}$ evaluation	<1 ms	∼5 min
100-point geometry sweep	0.3 s	∼8 h
12×100 material-geometry sweep	3 s	∼4 days
20-bead batch with statistics	5 s	Not available ^*a*^
In-app FEM solve (fine mesh)	2 s	Not applicable ^*b*^

^*a*^ Batch statistics were not run in the companion COMSOL study, so no timing is available. ^*b*^ COMSOL has no in-app equivalent of this cross-check, so no comparison time exists.

## Data Availability

The μFlow application (v1.0) is publicly and freely accessible at https://uflow.studio (accessed on 21 August 2026); it runs in any modern web browser and requires no installation, registration, or license. The material, bead, and benchmark parameters are given in Table 1, Table 2 and Table 3 and the noise parameters in Table 6. The interactive outputs of Section 3 (the batch statistics of Table 8 and the in-app FEM cross-check of Section In-App 2D Axisymmetric FEM Solver) are reproducible directly from the deployed application; the benchmark, sweep, and noise results (Table 4, Table 6, and Table 7; Figure 4, Figure 5, Figure 6, Figure 7 and Figure 8) were produced with a value-computation script that evaluates the same equations at the 16×16×8 quadrature of Section 3.1. The underlying source code and the value-computation script are available from the corresponding author on reasonable request. No experimental datasets were generated for this study.
